# Lagged Influence of Fine Particulate Matter and Geographic Disparities on Clinic Visits for Children’s Asthma in Taiwan

**DOI:** 10.3390/ijerph15040829

**Published:** 2018-04-23

**Authors:** Lung-Chang Chien, Yu-An Chen, Hwa-Lung Yu

**Affiliations:** 1 Epidemiology and Biostatistics, Department of Environmental and Occupational Health, University of Nevada, Las Vegas, NV 89154, USA; lung-chang.chien@unlv.edu; 2 Department of Bioenvironmental Systems Engineering, National Taiwan University, Taipei 10617, Taiwan; tmu536@gmail.com

**Keywords:** PM_2.5_, children’s asthma clinic visits, nonlinear lagged effects, spatial variation

## Abstract

Recent studies have revealed the influence of fine particulate matter (PM_2.5_) on increased medication use, hospital admission, and emergency room visits for asthma attack in children, but the lagged influence of PM_2.5_ on children’s asthma and geographic disparities of children’s asthma have rarely been discussed simultaneously. This study investigated the documented diagnosis of children’s asthma in clinic visits for children aged less than 15 years old that were associated with PM_2.5_ in two counties located in west-central Taiwan during 2005–2010. The result shows that PM_2.5_ had a significant lagged effect on children’s asthma for up to 6 days. A significantly higher relative risk for children’s asthma was more likely to happen at 2-day lag compared to the present day when PM_2.5_ increased from 36.17 μg/m^3^ to 81.26 μg/m^3^. Considering all lagged effects, the highest relative risk for children’s asthma was 1.08 (95% CI = 1.05, 1.11) as PM_2.5_ increased as high as 64.66 μg/m^3^. In addition, geographic disparities of children’s asthma were significant, and 47.83% of areas were identified to have children vulnerable to asthma. To sum up, our findings can serve as a valuable reference for the implementation of an early warning to governmental agencies about a susceptible population of children.

## 1. Introduction

Asthma is a common chronic disease in childhood and has become an increasing problem in the last few decades because numerous studies have revealed an increasing trend of asthma prevalence in both developed and developing countries [1,2,3,4,5,6,7,8]. In Taiwan, children’s asthma is a major health issue, and evidence has shown that the prevalence of children’s asthma has also increased dramatically over the last 40 years [9,10]. The overall 8-year prevalence of asthma in children was 15.7% according to a national survey from 2000 to 2007 [11]. Although the factors behind the changing pattern remain unclear, many personal and environmental factors have contributed to asthma risk, such as tobacco smoke, chemical fumes, weather conditions, and air pollution [12,13,14,15,16]. In addition, many industrial factories in Taiwan, especially in west-central Taiwan, play an important role in the process of economic development, but they contribute to various kinds of pollutants to the ambient air, which may trigger the increase of asthma risk [17].

Of the environmental factors, air pollution is one of the widespread environmental threats to human health. In particular, the issue of the association between air pollutants and asthma has been extensively discussed. The literature has proven that air pollution is a significant factor of asthma exacerbations, especially in susceptible populations who may be at risk for exacerbation of asthma from the toxic effects of airborne particulate matter (PM) [18,19,20,21]. Potential pathways for pulmonary effects propose that air pollutants can bypass the body’s natural defenses and provoke an inflammatory response in the lungs, especially from fine particulate matter (PM_2.5_, particulate matter with an aerodynamic diameter ≤2.5 μm) [22].

Several studies have revealed the influence of PM_2.5_ on increased medication use, hospital admission, and emergency room visits for asthma attack in children [23,24,25,26,27,28]; however, some studies found nonsignificant impacts of PM_2.5_ on asthma hospitalization [29,30]. Current studies have no clear explanations for the conflict in this regard [29,30]. The reasons may include variability in population characteristics and geographic variation. Some researchers have pointed out the space-time variation of PM composition and concentrations [31], which could partially explain the spatial differences in the health effects for mortality and morbidity [32]. The same levels of PM_2.5_ may still have different chemical compositions, which result in various adverse health effects. Epidemiological studies have demonstrated a strong spatial variation in the chemical component of PM that causes geographic disparities in human health [14,32,33,34,35].

The time-series studies in PM_2.5_ have found out the temporal pattern of associations between daily counts of health end-points and daily concentration on the current and a few preceding days [34,36,37]. Such lagged effects caused by PM_2.5_ have been observed in asthma and other respiratory symptoms, such as cough and wheeze, while the results are different from 3 to 4 days [38,39] or 2–6 days [40,41,42]. One possible reason for the different lags affected by PM_2.5_ is because different lagged effects may provide insight into different biologic mechanisms of reaction to PM_2.5_ [43]. Moreover, earlier research applied linear modeling approaches to assess the lagged effect between ambient air pollution and health effects [41,44,45]. However, the choice in the number of lags was not objective in different models, and the linear relationship between a health outcome and a lag is also doubtful. Recently, more studies have applied nonlinear models and reached more solid findings [14,46,47,48].

In this study, we hypothesized that the health impact of PM_2.5_ on asthma has a nonlinear lagged influence and that the district level of PM_2.5_ concentration might have different effects on asthma incidence. Thus, we adopted a spatiotemporal model for investigating the nonlinear relationship between PM_2.5_ concentrations and children’s asthma clinic visits with consideration of spatial autocorrelation and lagged effects. Our research aims comprise: first, evaluating the lagged effects of the daily PM_2.5_ concentration on the morbidity of asthma; and second, investigating geographic disparities of asthma risk after controlling for air pollutants and weather conditions.

## 2. Materials and Methods

### 2.1. Asthma Data

The data used in this study were retrieved from claim files of the National Health Insurance Research Database (NHIRD) provided by the Department of Health at the Bureau of National Health Insurance and managed by the National Health Research Institutes (NHRI). The NHIRD provides all inpatient and ambulatory medical claims for around 99% of Taiwanese residents [49]. With the approval of the NHRI, this study used the annual ambulatory care visit registry to claim data between 2005 and 2010, which provide information on the date of the clinic visit, up to three diagnoses, scrambled identification numbers of both patients and attending physicians, date of birth of patients, and gender. Asthma cases younger than 15 years old from 2005 to 2010 with the International Classification of Diseases, 9th Revision, Clinical Modification code from 493.00 to 493.99 were recruited in the study sample.

### 2.2. Environmental Data

**Figure 1:**
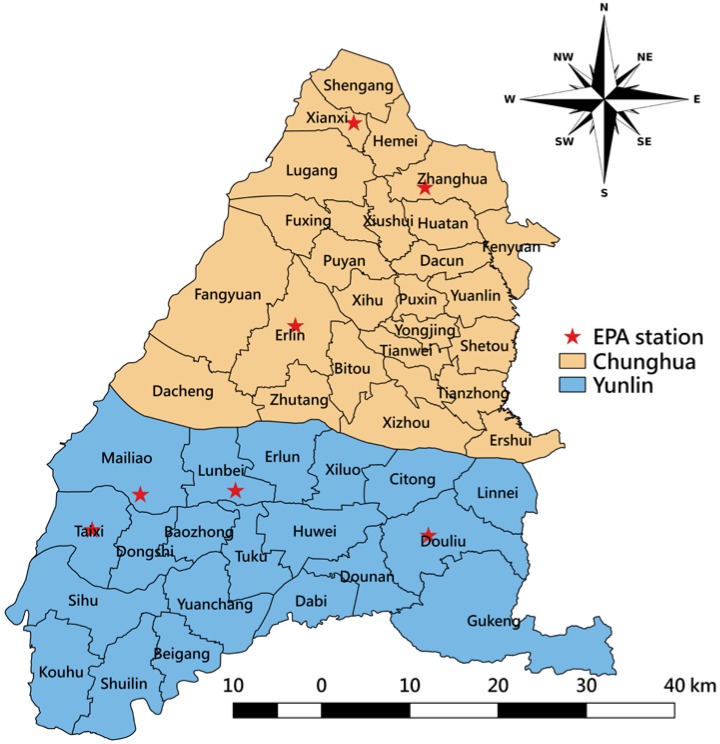
Map of the study area with 20 districts in Yunlin County and 26 districts in Changhua County. EPA = Environmental Protection Administration.

Measurements of air pollutants were based on data routinely collected at seven monitoring stations maintained by the Environmental Protection Administration in Taiwan (TWEPA): three in Changhua County and four in Yunlin County (Figure 1). Each monitoring station gathered hourly concentration data of carbon oxide (CO), nitric oxide and nitrogen dioxide (NO_x_), ozone (O_3_), sulfur dioxide (SO_2_), particulate matter with a diameter less than 10 μg/m^3^ (PM_10_), and PM_2.5_ together with weather-condition-related data, such as temperatures, relative humidity, wind speed, and wind direction. The Central Weather Bureau also provides weather records (temperature, relative humidity, and wind direction) from 35 weather monitoring stations in Changhua County and Yunlin County.

### 2.3. Study Area

Yunlin County is located in west-central Taiwan and accommodates one power plant, which is the third-largest coal-fired power plant in Taiwan. The widest east-west distance is about 50 kilometers (km) and the longest south-north distance is about 38 km. It has an area of 1291 km^2^ and a population of 710,000; 16% of the inhabitants are children. Changhua County is located at the center of Taiwan and is bordered by Yunlin County to the south. It has an area of about 1074 km^2^ and a population of around 1.3 million, of whom more than 17% are children. This county has a density of 7.7 factories per square kilometer. According to insurance administrative divisions, a total of 46 districts are contained in the study area, with 20 districts in Yunlin County and 26 districts in Changhua County as shown in Figure 1.

### 2.4. Imputation of Missing Environmental Data 

We used the Bayesian maximum entropy (BME) method to estimate the spatiotemporal distribution of air pollution concentrations and weather conditions for each unmonitored location by day from 2005 to 2010. The BME method is a spatiotemporal interpolation technique to incorporate measurement data, prior knowledge of neighbor information, and local spatiotemporal covariates [50,51,52,53] and has been applied to estimate the ambient pollution concentration across space–time previously [54,55,56,57]. The process of spatiotemporal air pollutants can be characterized by spatiotemporal trend and covariance. Nested spatiotemporal covariance models were used to characterize the spatiotemporal dependence of the air pollutants to reveal the spatiotemporal processes at different space-time scales [50,57]. Before performing spatiotemporal data imputation, the wind data were decomposed into two wind components along longitude and latitude, respectively, i.e., μ and ν, by using the formula μ = (wind speed) × sine (wind direction) and ν = (wind speed) × cosine (wind direction). The imputed wind data were reconstructed from the estimated μ and ν at unmonitored spatial locations and time instants.

### 2.5. Statistical Modeling Approach

This study adopted a distributed lag nonlinear model (DLNM) to investigate the nonlinear lagged influence of PM_2.5_ on children’s clinic visits for asthma. Suppose Y_it_ is the number of children’s asthma clinic visits at day t in district i, where Y_it_ follows a Poisson distribution with a mean parameter μ_it_. In order to adjust for the possible bias from the overdispersion problem, we especially considered a quasi-Poisson link function in the DLNM: log(μ_it_) = α + β × (DOW) + γ × (AP) + δ × (WD) + f(TP) + f(RH) + f(Time) + f(PM_2.5_, lag = 7) + f_spat_ + offset, where DOW is a day-of-the-week vector from Monday to Saturday (i.e., Sunday is the reference level), AP is a co-pollutant vector containing condensable particulate matter (cPM), the ratio of carbon oxide and nitrogen oxide (CO/NO_x_), SO_2_, and O_3_, and WD is an eight-level wind direction variable in terms of southwest (202.5–247.5°), west (247.5–292.5°), northwest (292.5–337.5°), north (337.5–22.5°), northeast (22.5–67.5°), east (67.5–112.5°), and southeast (112.5–157.5°), while south is a reference level (157.5–202.5°). We added three natural cubic splines for mean temperature (TP) with 6 degrees of freedom, mean relative humidity (RH) with 6 degrees of freedom, and calendar time (Time) with 21 degrees of freedom. The model also included a cross-basis function for PM_2.5_ to investigate the nonlinear association among children’s asthma, PM_2.5_, and lagged day. The cross-basis function is a 2-dimensional function from a natural cubic spline of PM_2.5_ with 4 degrees of freedom interacting with another natural cubic spline of lag up to 7 days with 3 degrees of freedom. A spatial function f_spat_ was included by using the Markov random fields (MRF) to take spatial autocorrelation into account [58]. The MRF can transform a shape file into a neighborhood matrix to depict whether any couple of areas share any piece of their boundaries. The number of neighboring districts in each district was also used to estimate unknown parameters of the MRF. The last term is an offset from the logarithm of the district population from 2005 to 2010.

All linear estimates derived from the model can be transformed into the relative risk (RR) by an exponential function. In particular, the linear estimates of air pollutants were transformed into the increased percentage of the relative risk (RR%) by an inter-quartile range change. The estimates of the cross-basis function can be also transformed into RR along with PM_2.5_ concentrations and lagged days. We defined 35 μg/m^3^ as the reference level of PM_2.5_ according to the PM_2.5_ standard published by TWEPA, which suggests less outdoor physical activity among at-risk individuals sensitive to respiratory diseases when the density of PM_2.5_ is over 35 μg/m^3^. The reference level of the lagged day is the present day (lag 0). The MRF can conduct a spatial estimate in each district, which can be also transformed into RR to explain the excessive asthma risk of each district compared with the average asthma risk from all districts.

Our data were maintained and managed by SAS v9.3 (SAS Institute, Cary, NC, USA), and missing data were imputed by using SEKS-GUI v.1.0.3 (SpaceTimeWorks, LLC, San Diego, CA, USA) [59]. DLNM estimation was performed with software R, version 3.1.2 (R Development Core Team, Vienna, Austria). The statistical significance of an estimate was determined by the 95% confidence interval (CI).

## 3. Results

**Figure 2:**
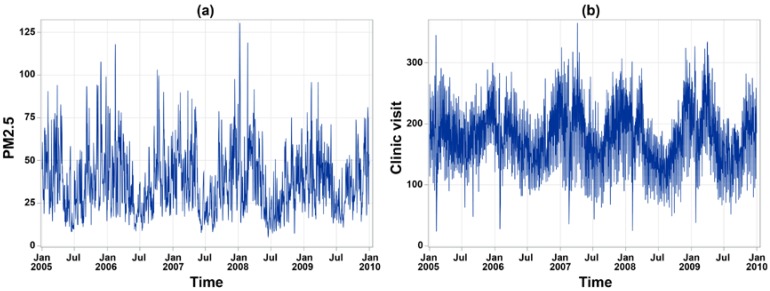
Trend plots of (**a**) daily PM_2.5_ concentration and (**b**) daily clinic visits for children’s asthma averaged by district from 2005 to 2010.

**Figure 3:**
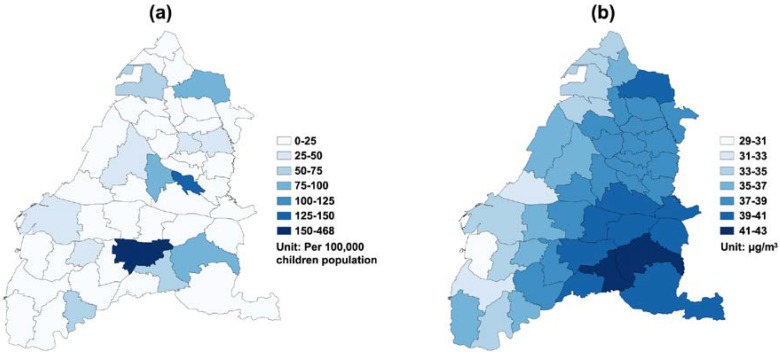
Geographical distribution of the (**a**) crude asthma visit rates (/100,000 children) and (**b**) daily average PM_2.5_ concentration (μg/m^3^) at the district level in Changhua County and Yunlin County from 2005 to 2010.

The number of annual clinic visits for children’s asthma was 172,696 in 2005, 157,278 in 2006, 162,837 in 2007, 148,439 in 2008, 150,680 in 2009, and 148,450 in 2010. The daily average of children’s asthma clinic visits was 4.70 cases (standard deviation (SD) = 14.28) in Yunlin County, while Changhua County only had 3.03 (SD = 7.94) children’s asthma clinic visits per day. Daily measurements of ambient pollutants and meteorological factors during the study period are summarized in Table 1. In particular, the mean daily average of PM_2.5_ was 37.22 μg/m^3^ (SD = 18.87) in Changhua County and 37.26 μg/m^3^ (SD = 19.38) in Yunlin County, and both were higher than the World Health Organization (WHO) guideline value (25 μg/m^3^). In addition, during the study period, 27.99% of days blow northeast wind in Changhua County, and 26.27% of days blow south wind in Yunlin County. Figure 2 shows a clear seasonality on the temporal variation of daily averaged PM_2.5_ concentration and daily clinic visits for children’s asthma. A total of 1543 days (70.4%) has the daily mean of PM_2.5_ concentration exceeding the WHO standard of 25 μg/m^3^ during the study period. On average, the study area had 180.22 daily clinic visits (SD = 51.84) for children’s asthma. A higher number of clinic visits more likely happened during springs and winters. Figure 3a depicts the geographic distribution of the average crude rate of daily children’s asthma and a higher rate more likely concentrated on a few eastern inland districts. Figure 3b presents the geographic distribution of daily average PM_2.5_ concentrations, in which there was a higher-density distribution from the western coast to the eastern inland districts in the whole study area, especially in the southeastern area.

Table 2 shows a significant RR of children’s asthma in each DOW compared to Sunday, whereas Monday had the highest RR by 2.07 (95% CI = 2.04–2.11; *p*-value < 0.0001). Moreover, compared to a southerly wind, a significantly lower RR was highly likely in a westerly wind (RR = 0.82; 95% CI = 0.76–0.89; *p*-value < 0.0001) and a northwesterly wind (RR = 0.89; 95% CI = 0.82–0.97; *p*-value = 0.0076). Only a northerly wind was significantly positively associated with children’s asthma (RR = 1.03; 95% CI = 1.00–1.06; *p*-value = 0.0272). Furthermore, increased asthma visits were significantly associated with cPM and SO_2_. When cPM increased one interquartile range (=17.60 μg/m^3^), the RR for children’s asthma significantly increased 1.42% (95% = 1.01–1.83; *p*-value < 0.0001). Similarly, when SO_2_ increased one interquartile range (=1.66 ppb), the RR significantly increased 1.25% (95% CI = 0.43–2.08; *p*-value = 0.0028).

**Figure 4:**
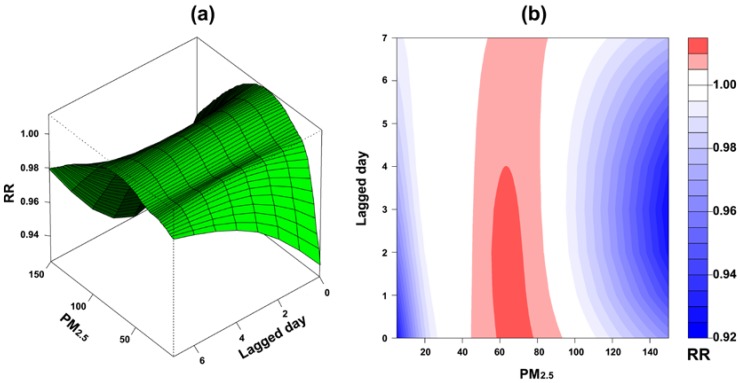
Transformed estimated cross-basis function for PM_2.5_: (**a**) The three-dimensional plot shows the variation of relative risk (RR) of children’s asthma along with PM_2.5_ concentration and lagged day; (**b**) The contour plot shows the hot spot of RR of children’s asthma in a coordinate of PM_2.5_ concentration and lagged day.

Figure 4a demonstrates the effect of PM_2.5_ concentration changes on children’s asthma along with lagged days, suggesting a higher RR of children’s asthma simultaneously increased when lagged day and PM_2.5_ concentration also increased. However, in each lagged day, the RR of children’s asthma gradually decreased when PM_2.5_ concentration increased over 80 μg/m^3^. A contour plot shown in Figure 4b presents a clear variation of RR by PM_2.5_ concentration and lagged day, indicating that a RR greater than 1 happened from present day and 4-day lag when PM_2.5_ concentration increased between 60 and 80 μg/m^3^.

**Figure 5:**
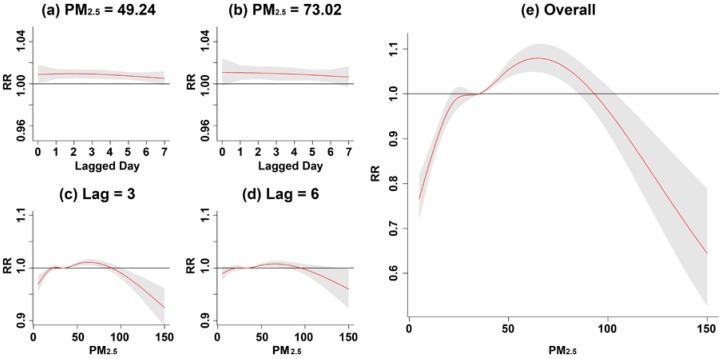
Overall relative risk and specific relative risk of children’s asthma with respect to selected PM_2.5_ concentrations and lagged days.

Compared to the reference level of PM_2.5_ (35 μg/m^3^), a higher concentration at the 75th percentile (49.24 μg/m^3^) and 95th percentile (73.02 μg/m^3^) of PM_2.5_ had a RR significantly higher than 1 from 1 to 6 lagged days as shown in Figure 5a,b. Compared to the present day, a higher RR was more likely to happen at 2-day lag, and it was significantly greater than 1 when PM_2.5_ increased from 36.17 μg/m^3^ to 81.26 μg/m^3^ (Figure 5c). The range of PM_2.5_ having a significant RR greater than 1 was shorter along with more lags. For instance, at 6-day lag, a significant RR greater than 1 can be only observed for the concentration of PM_2.5_ between 38.25 μg/m^3^ and 76.71 μg/m^3^ (Figure 5d). Figure 5e shows an overall PM_2.5_ effect on children’s asthma after accumulating all RRs from each lagged day along with PM_2.5_ concentration. The result displays a significant increase of RR when PM_2.5_ increased over 35 μg/m^3^. The cumulative RR reached the highest level by 1.08 (95% CI = 1.05, 1.11) as PM_2.5_ increased as high as 64.66 μg/m^3^. The increment of RR turned downward when PM_2.5_ was over 64.66 μg/m^3^, and no significant RR greater than 1 was observed when PM_2.5_ was higher than 85.25 μg/m^3^.

**Figure 6:**
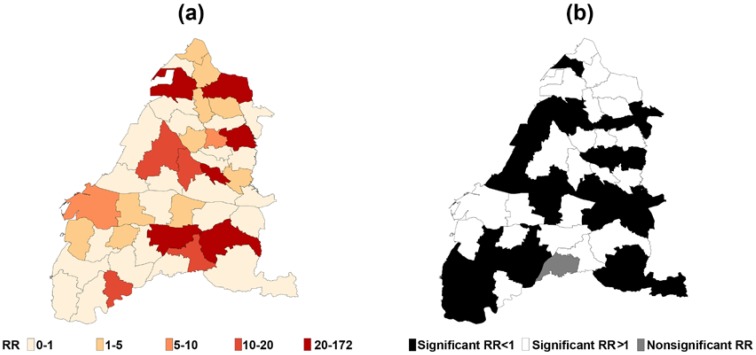
Maps for each district in Changhua County and Yunlin County according to the transformed estimated spatial function. Map (**a**) is the relative risk (RR) of children’s asthma from small (white) to large (red). Map (**b**) is the statistical significance of RR in each district determined by the 95% confidence interval, where black represents a significant RR < 1, white represents a significant RR > 1, and gray represents a nonsignificant RR.

Several districts were identified to have a higher RR for children’s asthma in Changhua County and Yunlin County. In Figure 6a, districts with a higher RR were more located in Changhua County, while the highest one was observed in the Huwei District (RR = 172.80; 95% CI = 162.83–183.39) at the center of Yunlin County, which is the second significant district for local industries, medical care, economy, and employment. Figure 6b reveals a total of 22 districts (13 in Changhua County and 9 in Yunlin County) with a RR significantly higher than 1 after controlling for the other confounding variables. Meanwhile, children living in 47.83% of the total 46 districts were vulnerable to asthma.

## 4. Discussion

Air pollutants, especially in PM_2.5_, have been implicated as a potential risk factor for human health, and have raised the greatest public health threat globally according to the WHO’s report [60]. This study selected children as the study population because children are very sensitive to air pollution. We investigated the exposure-lag-response association between children’s asthma and PM_2.5_ within the lag period, resulting in significantly lagged effects on children’s asthma, especially from 2-day lag to 6-day lag. In addition, this study revealed several high-risk districts after controlling for ambient air pollutants and weather conditions. The finding not only verified the evidence of geographic disparities on children’s asthma, but also provided a priority order of at-risk areas for advanced interventions or preventions.

The main feature of this study is taking the nonlinear properties into account for assessing associations between children’s asthma clinic visits and exposure to ambient air pollution. While some previous studies have examined the linear association between PM_2.5_ and asthma [23,24,25,61,62], actually at-risk children may not have any asthma symptoms during the concurrent day when being exposed to air pollutants. Meanwhile, the admissions observed on a particular day can be related to the air pollution observed on previous days. Therefore, using a linear model cannot reflect the true relationship of PM_2.5_ and health, and the lag itself may not be linearly correlated as well. The more lags we consider, the more nonlinearity among lags should be explicit.

Previous epidemiological research on asthma has usually defined lagged effects as linear terms in a model [25,26,27,28], while this modeling strategy assumed that those lagged terms were linearly independent with each in nature. This study adopted the DLNM, which is more flexible in defining the relationships among lags and emphasizes the interactions between PM_2.5_ concentrations and lags. Thus, the linearity assumption is no longer needed. In fact, recent studies had applied the DLNM more frequently to research lagged effects of air pollution on asthma. For instance, a Sweden study used this model to analyze air pollution data on primary health care visits for asthma, resulting in a significant finding from NO_2_ [63]. A similar application with the same model in Hong Kong evaluated the association of asthma emergency room visits among children with ozone concentration [64]. More importantly, our model includes a spatial function to adjust for spatial heterogeneity among 46 districts. The rationale of including the spatial function is to present a possible phenomenon known as harvesting, which appears as a raised risk ratio at a short lag followed by an apparent protective effect at a longer lag [65]. In other words, without including the spatial function in the DLNM, the effect of harvesting may be ignored, and may cause a monotone increasing trend in a disease risk as the concentration of an air pollutant increases. The situation has been well-investigated and discussed in a previous study of acute respiratory disease and PM_2.5_ [66].

We explored the temporal lag patterns of the effects of PM_2.5_ concentrations on children’s asthma clinic visits to conclude that PM_2.5_ was correlated with 1–6-day lags. Among the 6-day lagged effect, the first 3 days have the greatest relative risks. Consistent with our results, previous studies have suggested the lagged effect on different asthma outcomes to be at most 6 days. For instance, Ko et al. reported stronger lagged effect estimates from lag of 0–4 days for asthma hospitalization in Hong Kong [67]. Slaughter et al. examined the relationship between PM_2.5_ and asthma attack in children and found a significant effect for 0–1 day lag [25]. A longer lagged effect, up to 5 days, was observed among children [40]. The possible mechanism leading to the lagged effect could partly be explained by inflammation in the alveolar region of the lung caused by the smaller particles in the pollutant mixture [68]. The efficient deposition of ultrafine particles has been shown to be able to penetrate deep into the lungs and in particular in subjects with asthma [69]. In addition to differences in biological mechanism, the different lagged effects could be attributed to patient behavior patterns. Several days are needed for exacerbation to become severe enough to lead to a clinic visit. This would explain why the increase in asthma clinic visits was delayed. The finding of the lagged effects of asthma from the exposure to high PM_2.5_ episodes provides an important reference for governmental agencies to assess the health effects of the PM_2.5_ events, which have occurred frequently in recent years throughout Taiwan, in terms of clinic visits and their associated economic costs from the national health insurance plan.

The short-term time effect should be considered as a confounding factor for clinic visits due to the fact that seeking medical treatment varies by the day of the week. In Taiwan, generally the open time in most hospitals is from Monday morning to Saturday afternoon. Therefore, people who need to see a doctor on Sunday would wait until the following Monday to be admitted. The situation reflects a higher RR on Monday in our model, which illustrates the temporal pattern of medical treatment associated with the short-term time effect.

Because of a variety of air pollutants generated by certain sources, such as transportation and industry, are the major sources of CO and NO_x_, having those co-pollutants as confounding factors in the model is necessary. However, the correlation between CO and NO_x_ is as high as 0.81, causing the collinearity problem in the model. Although no previous study has examined whether collinearity will affect the estimation on a cross-basis function in the DLNM, we still alternatively used a single predictor by the CO/NO_x_ ratio to avoid potential biases. In addition, the CO/NO_x_ ratio has been frequently used in air quality assessment, where a high value indicates that mobile sources are the predominant contributors of these two compounds, while a low value of rationality indicates that point sources contribute from industrial sources [70,71]. Thus, this should be an adequate replacement rather than using CO and NO_x_ separately in the same model.

The spatial function of our model identified a significant excessive asthma risk in 22 districts after controlling for confounding variables. The finding concluded that those districts may have other unobserved risk factors, while we can only examine why children living in those districts are more vulnerable to asthma than children living in the other districts. Those unobserved factors may attribute to socioeconomic deprivation, other unobserved pollutants, or uneven medical resources. In our study, we observed that the high RR regions are mostly those locations with major medical centers, such as Douliu, Huwei, Beikang, and the Mailiao Townships in Yunlin County. This suggests that the spatial disparity of medical resources can be an important confounding factor in the spatial distribution of clinic visits for asthma.

This study revealed the concentration-response relationship from the population-based clinic visits data. As a result, our finding can provide a solid background for a governmental agency to develop an effective strategy to mitigate the health effects from PM_2.5_ concentration. Two limitations should be noted regarding this study. First, this study was not able to adjust for potential confounders at the individual level, such as body mass index, exposure to environmental tobacco smoke, genetic information, and allergens, because no such information is available in the NHIRD. Second, we were unable to identify the composition of PM_2.5_ from monitoring stations because of differences in pollutant composition across space and time and different influences during specific exposure periods.

## 5. Conclusions

Ambient levels of PM_2.5_ are associated with children’s asthma clinic visits in Taiwan. The study results clearly show that PM_2.5_ had significant lagged effects of up to 6 days on children’s asthma. Those identified high-risk districts reveal where vulnerable children may live, and their parents need to be informed in advance when a high concentration of PM_2.5_ is measured. The findings in this research can be useful for forecasting children’s asthma clinic visits in the coming days, and therefore, our results can serve as a valuable reference for the implementation of an early warning to governmental agencies about susceptible populations.

## Figures and Tables

**Table 1:** Characteristics of study variables.

Study Variables	Changhua		Yunlin	
	Mean	Standard deviation	Mean	Standard deviation
Children’s asthma cases	3.03	7.94	4.70	14.28
PM_2.5_ (μg/m^3^)	37.22	18.87	37.26	19.38
O_3_ (ppb)	28.89	10.01	31.68	10.67
SO_2_ (ppb)	3.73	1.42	3.59	1.39
cPM (μg/m^3^)	24.20	17.82	28.76	21.00
CO/NO_x_ (ppm/ppb)	0.02	0.01	0.02	0.01
Temperature (°C)	22.33	4.85	21.87	4.69
Relative humidity (%)	68.02	8.00	69.67	7.09
	Frequency	Percent	Frequency	Percent
Wind direction				
South	15,371	26.98%	11,510	26.27%
Southwest	4164	7.31%	2262	5.16%
West	307	0.54%	226	0.52%
Northwest	136	0.24%	162	0.37%
North	1160	2.04%	1714	3.91%
Northeast	15,944	27.99%	11,186	25.53%
East	8429	14.80%	7073	16.14%
Southeast	11,455	20.11%	9687	22.21%

PM_2.5_ = fine particulate matter; O_3_ = ozone; SO_2_ = sulfur dioxide; cPM = condensable particulate matter; CO = carbon oxide; NO_x_ = nitric oxide and nitrogen dioxide.

**Table 2:** Percentage change of relative risk (RR) in children’s clinic visits for asthma from 2005 to 2010.

Variable	RR	95% CI		*p*-Value
Day of the Week				
Sunday	1.00	Reference		-
Monday	2.07	(2.04, 2.11)		<0.0001
Tuesday	1.75	(1.72, 1.79)		<0.0001
Wednesday	1.71	(1.68, 1.74)		<0.0001
Thursday	1.45	(1.42, 1.48)		<0.0001
Friday	1.64	(1.61, 1.67)		<0.0001
Saturday	1.74	(1.71, 1.77)		<0.0001
Wind direction				
South	1.00	Reference	-	
Southwest	1.00	(0.97, 1.02)	0.6907	
West	0.82	(0.76, 0.89)	<0.0001	
Northwest	0.89	(0.82, 0.97)	0.0076	
North	1.03	(1.00, 1.06)	0.0272	
Northeast	1.00	(0.98, 1.02)	0.9091	
East	1.00	(0.98, 1.02)	0.9532	
Southeast	1.00	(0.99, 1.02)	0.7150	
	RR%	95% CI	*p*-Value	
Air pollutant				
cPM	1.42	(1.01, 1.83)	<0.0001	
CO/NO_x_	−3.16	(−3.85, −2.46)	<0.0001	
SO_2_	1.25	(0.43, 2.08)	0.0028	
O_3_	0.82	(−0.07, 1.71)	0.0704	

Abbreviation: RR = Relative risk; RR% = Increased percentage of relative risk per interquartile range change; CI = Confidence interval.
